# Computerized virtual surgery based on computational fluid dynamics simulation for planning coronary revascularization with aortic root replacement in adult congenital heart disease: a case report

**DOI:** 10.1007/s11748-020-01517-w

**Published:** 2020-11-01

**Authors:** Yu Hohri, Keiichi Itatani, Sachiko Yamazaki, Hitoshi Yaku

**Affiliations:** Department of Cardiovascular Surgery, Cardiovascular Blood Flow Imaging Research Laboratory, Kyoto Prefectural University of Medicine, 465 Kajii-cho, Kawaramachi Hirokoji, Kamigyo-ku, Kyoto, 602-8566 Japan

**Keywords:** CFD simulation, Blood flow imaging, Computerized virtual surgery, Adult congenital heart disease, Supravalvular aortic stenosis

## Abstract

A 38-year-old woman presented with exertional dyspnea and chest compression. She had undergone repair of congenital supravalvular aortic stenosis at 8 years of age. Contrast-enhanced computed tomography showed re-stenosis in the ascending aorta, bilateral coronary arterial aneurysm, and a highly thickened left ventricular wall. Release of stenosis was necessary to avoid left ventricular functional deterioration; however, it could cause demand–supply mismatch in coronary flow due to substantial left ventricular hypertrophy. Sufficient statistical evidence was not available in this situation; therefore, computerized virtual surgery based on computational fluid dynamics (CFD) was performed to predict the postoperative hemodynamics. Consequently, root replacement with in situ Carrel patch coronary reconstruction was considered a better option than coronary artery graft bypass in the left-side coronary flow supply. The patient underwent root replacement with in situ Carrel patch coronary reconstruction as planned based on CFD without any complication and was discharged 15 days postoperatively.

## Introduction

Complicated anatomy and hemodynamics are often problematic in adult congenital heart disease (ACHD) patients due to the paucity of data regarding clinical experience and the short history of congenital heart surgery [1, 2]. In these patients, especially in those who require extraanatomical reconstruction, computerized virtual surgery with computational fluid dynamics (CFD) simulation, combined with 3D computer graphics (CG), plays a key role in surgical planning with accurate prediction of the postoperative hemodynamics [3].

## Case

**Fig. 1:**
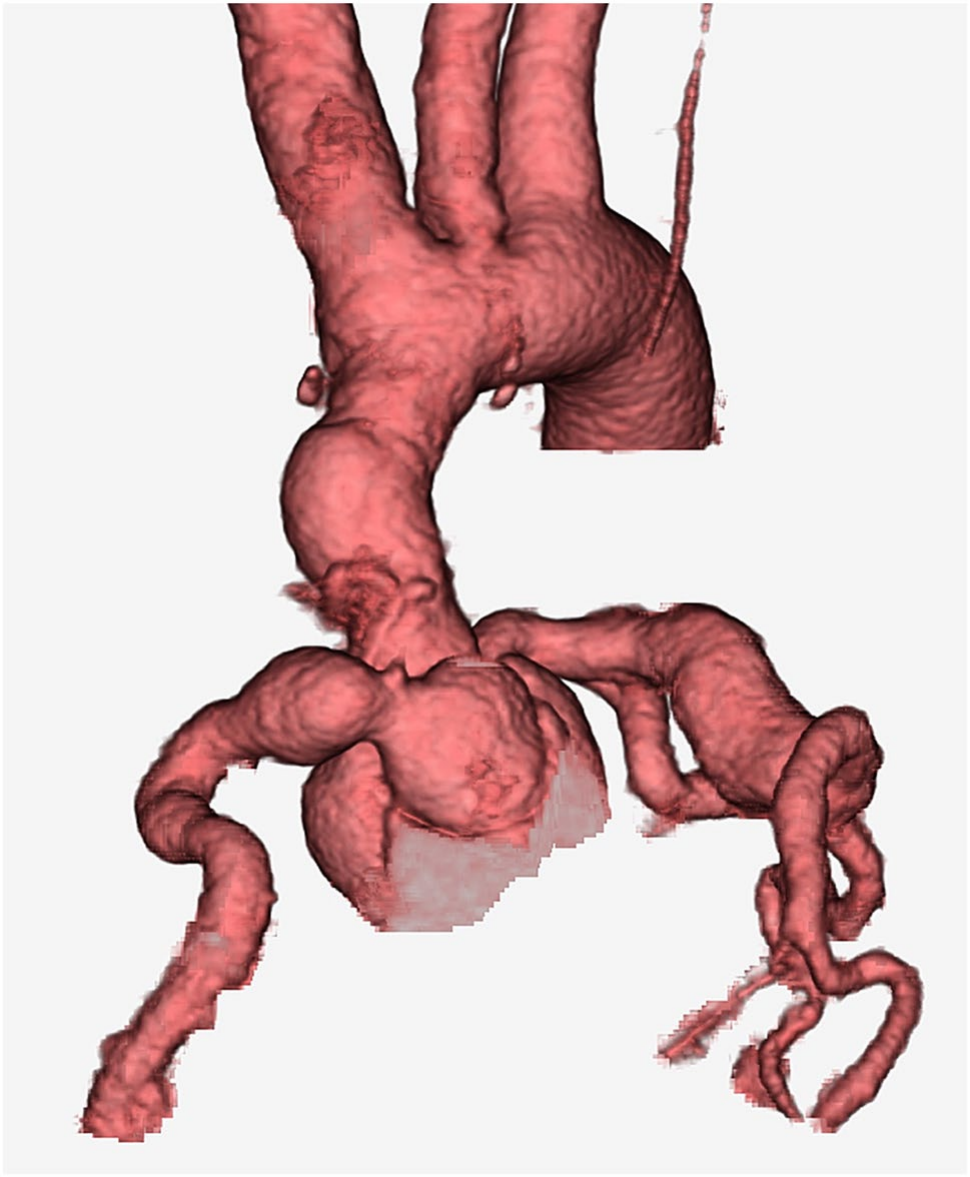
Preoperative CT image. CT imaging showed the supravalvular aortic stenosis and bilateral coronary aneurysm. *CT* computed tomography

**Fig. 2:**
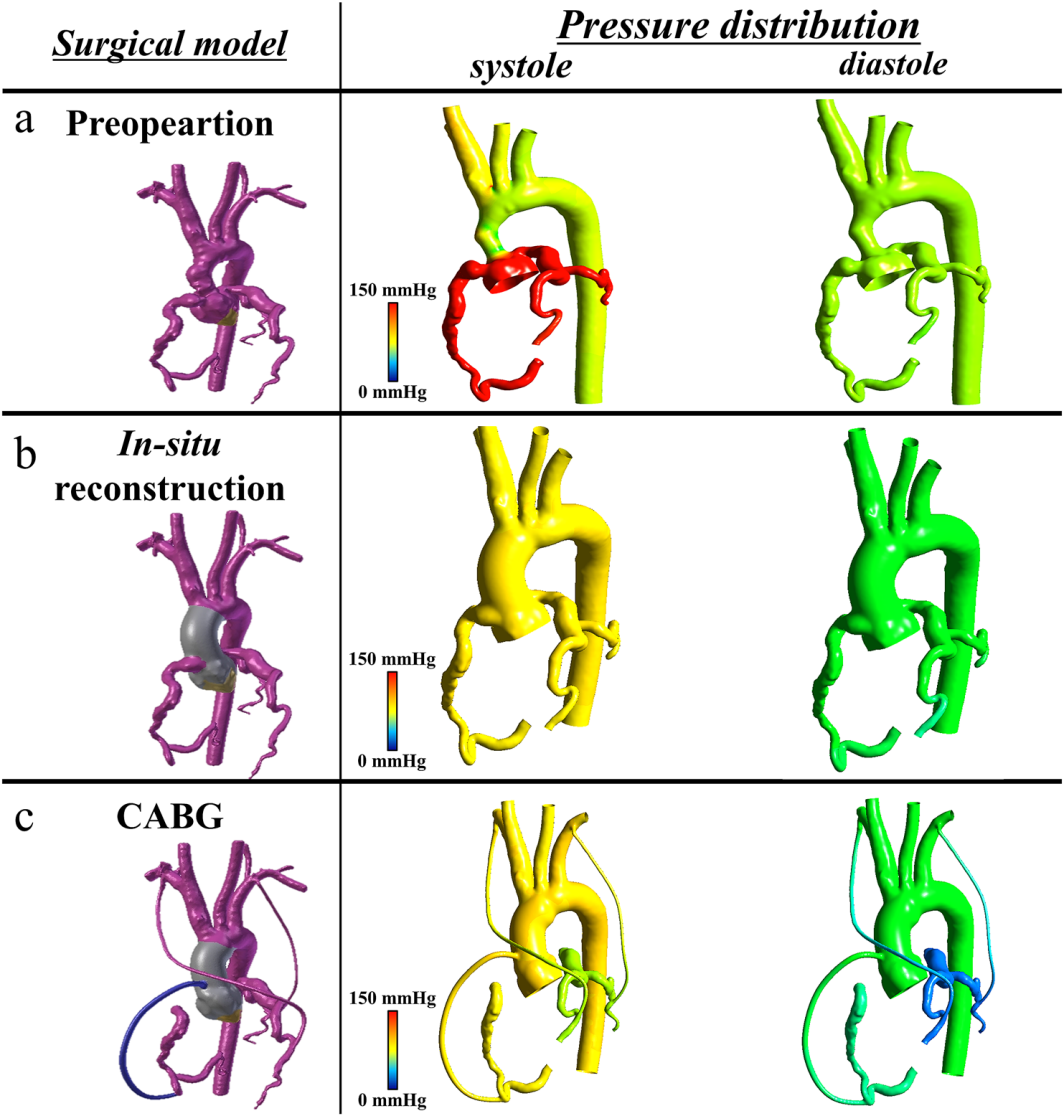
Computerized virtual surgery with CFD simulation. **a** Pre-operation. **b** In situ reconstruction with root replacement. **c** CABG with root replacement. *CABG* coronary artery graft bypass, *CFD* computational fluid dynamics

**Fig. 3:**
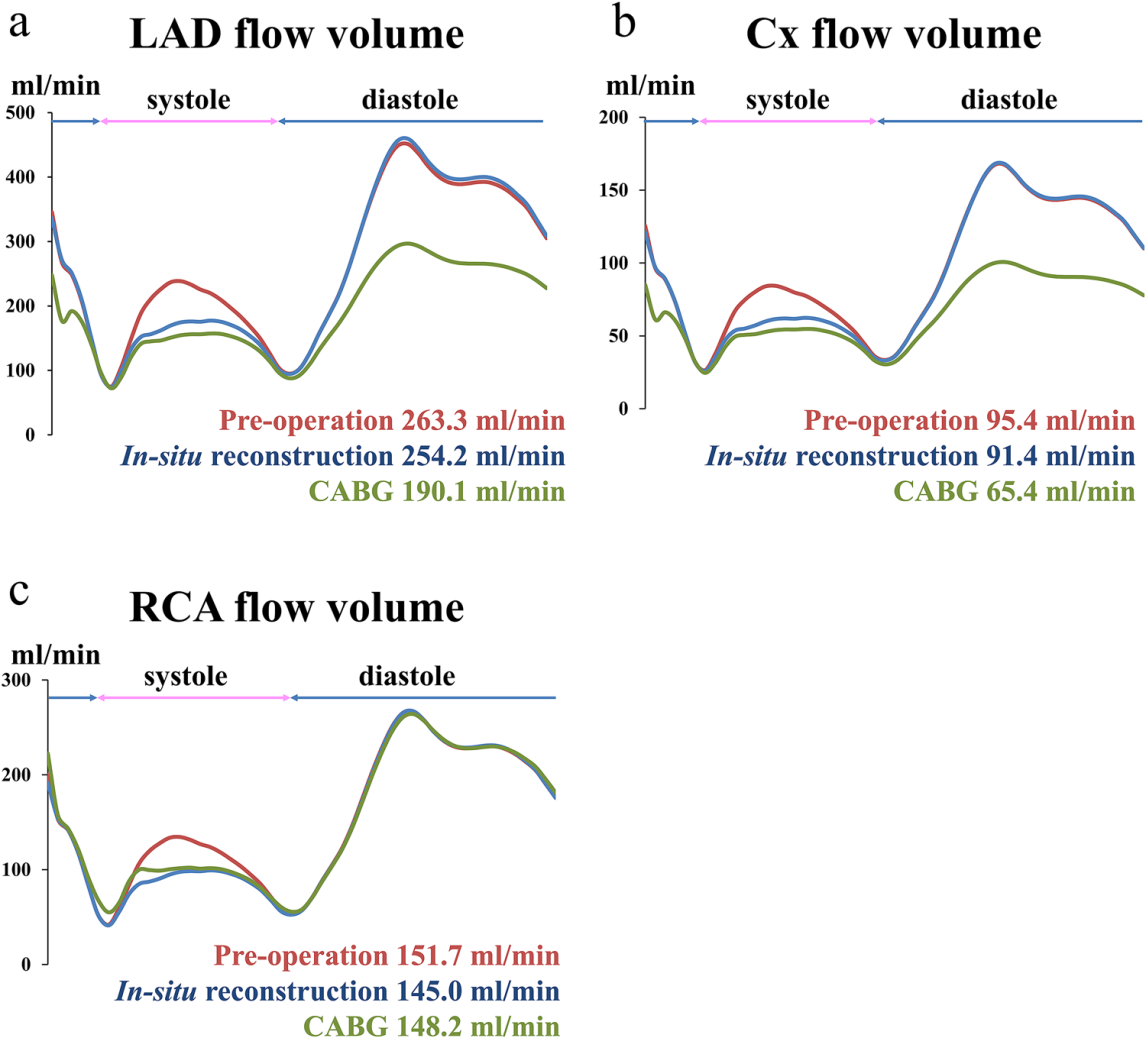
Coronary flow in various graft patterns during one cardiac cycle. **a** LAD, **b** LCx, **c** RCA. *LCx* left circumflex artery, *LAD* left anterior descending artery, *RCA* right coronary artery

A 38-year-old woman, who had undergone correction of congenital supravalvular aortic stenosis at 8 years of age, developed progressive exertional dyspnea and chest pain for more than a year prior to the current presentation. Electrocardiogram-gated contrast-enhanced computed tomography (CT) showed stenosis in the ascending aorta and highly dilated bilateral coronary arteries (Fig. 1). Echocardiography showed normal left ventricular (LV) wall motion with an ejection fraction of 59%, prominent LV hypertrophy, mild aortic regurgitation, and flow acceleration of 5.2 m/s in the ascending aorta. Thallium myocardial scintigraphy did not detect myocardial ischemia. Catheter examination demonstrated a pressure drop of 110 mmHg at the stenosis site of the ascending aorta with high pressure in the sinus of Valsalva. Three-dimensional time-resolved cine phase contrast magnetic resonance imaging (4D flow MRI) demonstrated preserved LV wall motion and cardiac output but highly accelerated flow (> 5.0 m/s) in the ascending aorta. Release of stenosis was necessary to resolve the patient’s complaint and avoid LV functional deterioration due to excessive afterload; however, when we had performed simply aortic root replacement with the in situ coronary revascularization during surgery, reduction of the blood pressure in the sinus of Valsalva may have led to demand–supply mismatch in coronary flow due to heavily dilated and tortuous coronary artery and the highly thickened LV wall muscle. Moreover, we also suspected to harvest dense adhesion around the sinus of Valsalva due to the old patch material in this reoperation. Therefore, we had to examine whether CABG could be a candidate or alternative option for coronary reconstruction. However, we could not determine which strategy of revascularization could supply exactly more sufficient coronary perfusion, CABG or in situ reconstruction with the Carrel patch technique. Therefore, we performed computerized virtual surgery based on CFD combined with 3D CG. The CFD method was based on our previous validation studies [4, 5]. We used the data obtained from thin-slice, early-phase, enhanced multidetector-row CT imaging. These imaging data were transformed to a 3D patient-specific geometry by Osirix (Osirix Foundation, Geneva, Switzerland) and 3D-Coat (PIGWAY, Kiev, Ukraine). Computational meshes were created with ANSYS-ICEM CFD 16.0 (ANSYS Japan, Tokyo, Japan). To simulate the blood flow, the inlet boundary conditions in the aortic root were set as the mass flow boundary condition with a pulsatile wave. Cardiac outputs were set at 5.0 l/min based on the cardiac catheterization data. The outlet boundary conditions were used as the pressure boundary conditions that represented the external forces outside of the analysis domain. The physiological external forces in the neck vessels and the descending aorta were composed of the reflection wave from the peripheral tissue, vascular inertance, and autonomic regulation. In coronary artery outlets, time-varying impedance that expressed the systolic ventricular muscle contraction and diastolic relaxation, can realize perfused muscle volume or peripheral vascular bed capacity, as reported previously [3, 6]. Finite volume method with backward Euler transient calculation was used to solve the Navier–Stokes equation with the convergence criteria of 1.0E-5 for all parameters. Calculated flow velocity distribution inside the aorta resulted in almost the same flow map as that detected in the 4D flow MRI. The calculated pressure drop in the ascending aorta was high and almost the same as that measured in the catheter examination. The computerized virtual postoperative models with 3D CG were of two types: (1) in situ coronary reconstruction with Carrel patch technique for root replacement; (2) CABG procedure using the bilateral internal thoracic artery (ITA) to the left anterior descending artery and circumflex branch and saphenous vein graft from the ascending aorta to the right coronary artery (RCA) in addition to root replacement. The predicted postoperative coronary flow supply is illustrated in Fig. 2. The shortage of blood supply to the left-side coronary arteries was detected in the CABG model despite using the bilateral ITA. Compared with the preoperative state, in situ coronary reconstruction with the Carrel patch technique decreased the coronary blood flow in the systole due to the reduction in the Valsalva pressure but obtained sufficiently increased coronary flow in the diastole because of the increased intensity of reflection wave from the peripheral vasculature (Fig. 3).

Based on these results, we performed aortic root replacement using a 24-mm Valsalva graft (Japan Lifeline Co. Ltd., Tokyo, Japan) implanted with a 21-mm bi-leaflet mechanical valve (St Jude Medical, St Paul, Minnesota, USA) with in situ coronary reconstruction using the Carrel patch technique. Before surgery, we planned to perform the valve-sparing aortic root replacement as possible. However, during surgery, we observed the severely degenerated and thickened aortic leaflet with the degeneration of the sinus of Valsalva. Thus, because these aortic leaflets could not be expected for long-term durability, we determined that the aortic valve should be replaced. The patient was discharged 15 days postoperatively without any complications in the coronary arterial system. A postoperative electrocardiogram revealed non-ST elevation. Moreover, echocardiography and contrast-enhanced CT showed preserved LV function and sufficiently widened ascending aorta without coronary arterial stenosis or thrombosis, respectively. Two years after the surgery, she was well with no angina symptoms.

## Discussion

In the present case, performing computerized virtual surgery with CFD simulation could help the surgeon predict the postoperative coronary flow volume and help determine the surgical strategy for coronary aneurysm in supravalvular aortic stenosis.

Coronary abnormality is one of the major complications of supravalvular aortic stenosis in adults [7, 8]. Coronary arteries may be dilated and tortuous because aortic root hypertension causes enhanced intimal thickening and accelerated atherosclerosis [8]. However, because only a few cases have been reported, Yilmaz et al. mentioned that the decision to undertake surgical repair for coronary aneurysms depends on the surgeon’s experience [8]. In the updated American College of Cardiology and American Heart Association guidelines, clinical recommendations for coronary revascularization are not provided [9]. Therefore, it is difficult to plan the surgical strategy in cases of coronary aneurysm with supravalvular aortic stenosis.

In the present case, the coronary flow demand–supply mismatch was a concern due to heavily dilated and tortuous coronary after the release of supravalvular aortic stenosis. Large diameter vessels do not always provide sufficient flow volumes, especially when turbulent flow is expected. Thus, in this particularly rare case, we were not sure which surgical reconstruction methods of coronary artery supply stable blood flow to the myocardium. Moreover, it was difficult to compare the coronary blood volume accurately between CABG and in situ reconstruction, because coronary blood distribution, the inflow blood volumes and perfusion timing are different between these surgical procedures, and the coronary arterial impedance is constantly changing during the cardiac cycle due to ventricular muscular contraction and relaxation. Thus, because various hemodynamic conditions are different, it is not easy to compare the coronary flow volume in both surgical procedures. Therefore, we determined the strategy of coronary revascularization by performing virtual surgery with CFD simulation. CFD simulation can help determine the blood flow pattern in small vessels, such as coronary arteries, because of high temporal and spatial resolution. Furthermore, in the present case, to simulate the blood flow that would be as similar as possible to the actual blood flow, we used well-validated outlet boundary conditions that represented physiological phenomena including peripheral reflection, vascular inertance, autonomous regulation, and coronary perfusion capacities [10]. As a result, we could predict that in situ Carrel patch coronary reconstruction would be superior to CABG in terms of the postoperative volume of left coronary flow supply. Thus, preoperative computerized virtual surgery with reliable prediction of postoperative hemodynamics is advantageous for the determination of surgical procedures, especially in highly invasive surgical cases.

The major limitation of this report is that we could not include the postoperative change in physiological parameters in our CFD calculation; for example, in the present case, reduced myocardial volume due to reverse remodeling after the reduction of LV afterload. Furthermore, it is impossible to evaluate the material property of the vessel wall with the present technique. However, virtual surgery with CFD simulation is the only method to compare the postoperative coronary flow between CABG and in situ reconstruction; as a result, we believe that CFD simulation was useful to determine the appropriate strategy of coronary revascularization in the present case.

## Conclusion

The accuracy of CFD simulation has been reported in previous studies [11, 12]; however, to the best of our knowledge, the present study is the first report to use the computerized virtual surgery with CFD simulation for determination of coronary revascularization procedure. It is difficult to plan a strategy for complicated coronary revascularization due to the complicated anatomy and physiology of the region in ACHD. Computerized virtual surgery with CFD simulation can help to determine the optimal graft design because it can predict the volume of coronary flow in various graft patterns before the actual operation.
